# Electroencephalogram evaluation of accidental cerebral congestion during unexpected superior vena cava clamping: a case report

**DOI:** 10.1186/s40981-022-00531-6

**Published:** 2022-06-09

**Authors:** Hirotsugu Miyoshi, Ryuji Nakamura, Sachiko Otsuki, Yuko Noda, Noboru Saeki, Yasuo M. Tsutsumi

**Affiliations:** Department of Anesthesiology and Critical Care, Hiroshima University Hospital, 1-2-3 Kasumi, Minami-ku, Hiroshima, 734-8551 Japan

**Keywords:** Electroencephalography monitoring, Brain wave, Cerebral congestion, Superior vena cava clamping

## Abstract

**Background:**

Intraoperative superior vena cava (SVC) clamping causes hypotension and cerebral congestion. There is no established method for monitoring brain function during cerebral congestion. We encountered a case of cerebral congestion caused by unexpected SVC clamping.

**Case presentation:**

A 64-year-old man underwent SVC clamping during lung tumor resection. The entropy and electroencephalogram monitoring values decreased with SVC clamping and increased in response to the release of congestion by phlebotomy and SVC declamping.

**Conclusions:**

Because entropy sharply reflects brain viability during cerebral congestion, it was considered helpful in evaluation of the monitoring of cerebral congestion.

## Introduction

Unscheduled superior vena cava (SVC) clamping during surgery is life-threatening and challenging for anesthesiologists. Acute clamping of the SVC during surgery causes a decrease in the cardiac output and hypotension due to reduced venous return to the heart and reduces cerebral perfusion flow and cerebral congestion owing to increased central venous pressure (CVP). We need to decide on a case-by-case basis. There are no established management guidelines for cerebral congestion caused by unexpected SVC clamping, and appropriate monitoring of cerebral viability is necessary to avoid irreversible cerebral dysfunction. However, there is no consensus on monitoring cerebral perfusion or cerebral function during cerebral congestion.

Entropy™ (GE Healthcare, Helsinki, Finland) and Bispectral Index® (BIS; Medtronic-Covidien, Dublin, Ireland), which are processed electroencephalogram (EEG) monitors, are routinely used to evaluate the depth of anesthesia. With the entropy module, we can know the state entropy (frequency range from 0.8 to 32 Hz) calculated from the EEG, the response entropy (frequency range from 0.8 to 47 Hz) obtained by adding the electromyogram component to the SE, and the BSR value (%) indicating the ratio of flat EEG per minute. EEG has also been reported to be useful as an indicator of cerebral ischemia and cerebral hypoperfusion, and the entropy value calculated from EEG is expected to monitor cerebral function during cerebral congestion [1].

We report a case of cerebral congestion during an unexpected SVC clamping. This case showed that entropy is useful for evaluating brain viability and function during cerebral congestion. Written consent for publication was obtained from all patients.

## Case description

**Fig. 1:**
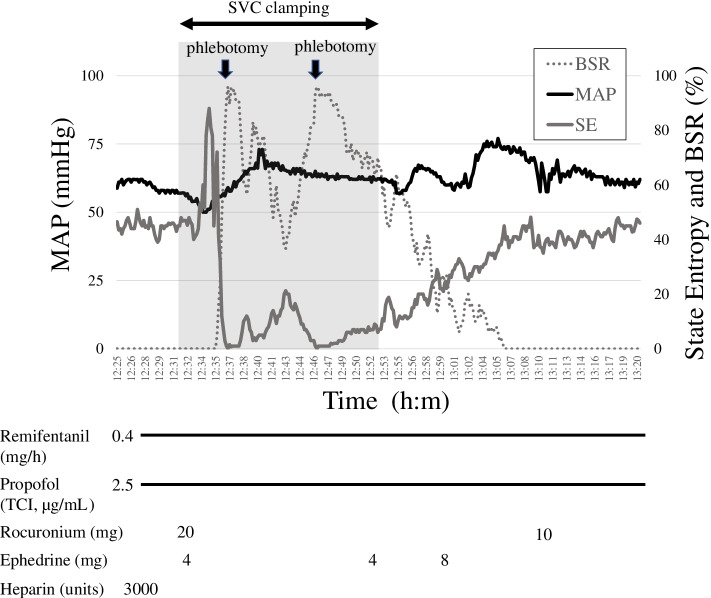
Clinical course. Black line indicates mean atrial pressure (MAP). The gray line indicates the state entropy (SE). The gray dotted line indicates the burst suppression ratio (BSR). The black arrow indicates phlebotomy. During cerebral congestion, the gray area indicates superior vena cava (SVC) clamping. Increases in SE and decreases in BSR were observed two times after the phlebotomy procedures. TCI, target-controlled infusion

**Fig. 2:**
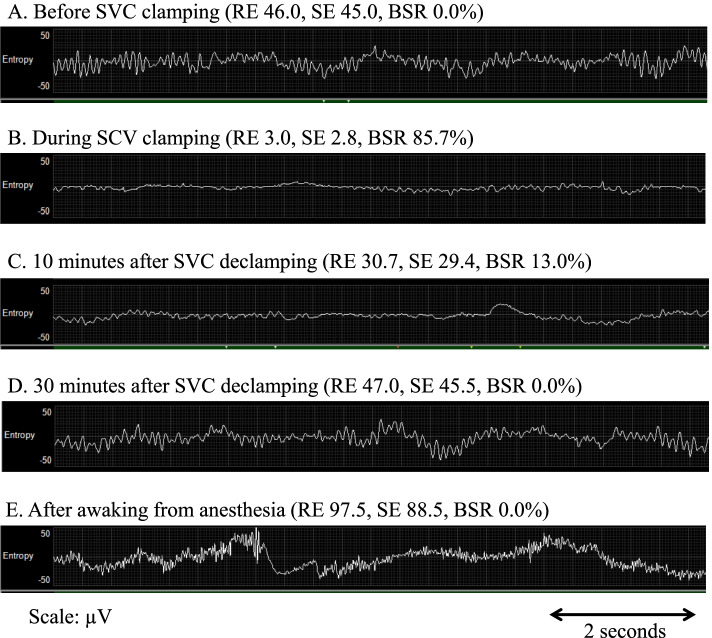
Electroencephalogram before and after superior vena cava (SVC) clamping. This figure shows the electroencephalogram before and after the SVC clamping. The vertical axis indicates amplitude (μV). The horizontal axis indicates the time, and the arrow scale means 2 s. **A** Before SVC clamping; α waves and Θ waves are mixed. **B** During SCV clamping; brain waves disappear and flatten. **C** Ten minutes after SVC declamping; brain waves remain flat. **D** Thirty minutes after SVC declamping; the brain wave recovers, and the waveform is the same as before the clamp. **E** After awaking from anesthesia; β wave, which is an awakening wave, appears. The values of response entropy (RE), state entropy (SE), and burst suppression ratio (BSR) are shown

A 64-year-old man underwent video-assisted thoracic surgery, right upper lobectomy combined with bronchoplasty, and SVC plasty for right lung cancer. General anesthesia was induced and maintained with propofol and remifentanil combined with paravertebral nerve block. After induction of anesthesia, propofol was administered at 2.5 μg/mL with a target-controlled infusion system (TCI pump TE-371TM; Terumo, Tokyo, Japan), and remifentanil was administered at 0.4 mg/h. Before clamping the SVC, the state entropy remained around 50 (Fig. 1). Because the tumor had invaded the SVC, it was necessary to clamp the SVC for resection. After administration of 3000 units of heparin, the SVC was clamped on the cranial and caudal sides of the tumor, and the invading tumor was excised simultaneously with the SVC. Immediately after clamping, the mean arterial pressure (MAP) dropped from 60 to 50 mmHg. Blood pressure quickly recovered to its previous level after administration of 4 mg of ephedrine. State entropy rose temporarily for 4 min after clamping and then steeply slowed down to a value less than 10, and the burst suppression ratio (BSR) increased from 0 to 93% (Fig. 2A and B). The flattening of EEG was thought to be caused by cerebral congestion and reduced cerebral perfusion; however, the clamp could not be released because the SVC was already incised. We performed phlebotomy by releasing the clamp on the cranial side to relieve SVC congestion. Time course of an increase of MAP, phlebotomy, increase of state entropy (SE), and decrease of BSR should be correctly described. SE increased after declamping SVC (Fig. 1). We performed phlebotomy with reference to the improvement in entropy EEG. Phlebotomies were performed twice during a total SVC clamping time of 21 min, for a total volume of 600 mL. State entropy gradually increased after SVC declamping and fully recovered 30 min after declamping (Fig. 2C, D, and E). After the surgery time of 7 h and 12 min, he awakened from anesthesia and was extubated in the operating room. The patient was discharged without neurological sequelae.

## Discussion

In the present case, unplanned SVC clamp during lung cancer surgery caused cerebral congestion and flattened EEG waves. Since the hemodynamic fluctuations after the SVC clamp were mild, we considered that cerebral congestion was also mild. However, as the EEG waves flattened rapidly, we needed to get rid of cerebral congestion. Considering the urgency, we tried phlebotomy instead of bypass surgery. As Fig. 1 shows, improvement in entropy (increased SE value and decreased burst suppression ratio) was consistent with the two phlebotomy procedures. These trends were not associated with improvement in hypotension with hypertensive drugs. In addition, propofol and remifentanil were administered continuously, and their infusion rate was not changed. From these facts, we determined that the change in entropy reflects the state of cerebral congestion caused by the SVC clamp.

In some animal model studies, brain function during SVC clamping can be maintained for a relatively long time if cerebral perfusion is maintained. Urayama et al. reported that neurological impairment was not observed after 120-min SVC clamp despite reduced amplitude and prolonged latency of somatosensory-evoked potentials during clamping in dogs [2]. Masuda et al. demonstrated no abnormalities in EEG with reduced regional cerebral blood flow to about 80% by SVC clamping for 60 min in monkeys [3]. Even from clinical data, it is considered that brain damage does not occur if the SVC clamp is within 45 to 60 min [4]. In a small-scale multicentric study, postoperative neurological complications did not occur with a median of 40-min clamping (including 100 min or more) [5]. Many studies have shown that cerebral congestion with SVC clamping is reversible, and cerebral viability can withstand long-term clamping if cerebral circulation is maintained.

For scheduled SVC clamping, it is common to decompress the SVC by veno-veno bypass to avoid cerebral congestion, which is used in cases of high venous pressure [6]. In accidental SVC clamping, the anesthesiologist must determine whether the patient can tolerate cerebral congestion. The measurement of CVP and regional oxygen saturation (rSO_2_) has been reported to evaluate cerebral congestion due to SVC clamping, but there is no established evaluation or management method. Furthermore, the assessment of cerebral congestion using these methods is indirect and does not evaluate brain viability. Both BIS and entropy are calculated based on EEG. Frequency of EEG waves is decreased in response to mild ischemia [7, 8], which is also induced by cerebral congestion [6], suggesting that entropy also decreased by cerebral congestion.

In a report using BIS in an SVC clamping, the value of BIS after declamping was proportional to MAP [1]. However, this report does not discuss the relationship between cerebral congestion state and BIS values. In the present case, the gradual increase in entropy after the release of cerebral congestion indicated an improvement in brain function. In the present case, a rapid decrease in entropy and a sudden rise in BSR were observed approximately 4 min after SVC clamping. We increased the blood pressure with ephedrine and depressurized CVP by phlebotomy. Increases in entropy and decreases in BSR were observed with two phlebotomy procedures. The timing of phlebotomy and recovery of entropy were the same. This clinical course assumes that entropy clearly reflects cerebral perfusion flow and brain viability during cerebral congestion due to SVC clamping. Raising blood pressure may be effective against cerebral congestion, but we cannot assess the effectiveness of raising blood pressure in this case because we did not attempt to raise the MAP above the normal range.

Entropy, the processed EEG monitor, has the possibility of reflecting brain viability during cerebral congestion and could be useful for evaluating cerebral congestion monitoring.

## Data Availability

The data used in this case report are available from the corresponding author on reasonable request.

## Abbreviations

SVC: Superior vena cava
CVP: Central venous pressure
EEG: Electroencephalogram
BIS: Bispectral index
MAP: Mean arterial pressure
BSR: Burst suppression ratio
rSO_2_: Regional oxygen saturation
TCI: Target-controlled infusion
